# Overexpression of B7-H3 in α-SMA-Positive Fibroblasts Is Associated With Cancer Progression and Survival in Gastric Adenocarcinomas

**DOI:** 10.3389/fonc.2019.01466

**Published:** 2020-01-10

**Authors:** Shenghua Zhan, Zhiju Liu, Min Zhang, Tianwei Guo, Qiuying Quan, Lili Huang, Lingchuan Guo, Lei Cao, Xueguang Zhang

**Affiliations:** ^1^Department of Pathology, Jiangsu Institute of Clinical Immunology, The First Affiliated Hospital of Soochow University, Suzhou, China; ^2^Department of Pathology, The First Affiliated Hospital of Soochow University, Suzhou, China; ^3^Department of Pathology, Children's Hospital of Soochow University, Suzhou, China; ^4^Department of Pathology, Changshu Hospital of Affiliated to Nanjing University of Chinese Medicine, Changshu, China; ^5^Department of Clinical Laboratory, Children's Hospital of Soochow University, Suzhou, China; ^6^Jiangsu Institute of Clinical Immunology, The First Affiliated Hospital of Soochow University, Suzhou, China; ^7^Jiangsu Key Laboratory of Gastrointestinal Tumor Immunology, The First Affiliated Hospital of Soochow University, Suzhou, China; ^8^Jiangsu Key Laboratory of Clinical Immunology, Soochow University, Suzhou, China

**Keywords:** cancer-associated fibroblast, B7-H3, α-SMA, gastric adenocarcinoma, prognosis

## Abstract

**Background:** B7-H3 promotes tumor immune escape and is highly expressed in tumor tissues. Stromal cells in tumors, including fibroblasts, play an important role in this process; however, the role of B7-H3 in tumor fibroblasts has not been fully clarified.

**Methods:** We examined B7-H3, CD31, and alpha-smooth muscle actin (α-SMA) protein expression in 268 gastric adenocarcinomas (GACs) by immunohistochemistry. The coexpression of B7-H3 with CD31 or α-SMA was examined using immunofluorescence double staining. Cytokine expression from fibroblasts treated with B7-H3 small interfering RNA (siRNA) was analyzed by a Quantitative reverse transcription-polymerase chain reaction (qPCR) and Enzyme-linked immunosorbent assay (ELISA). The transwell tests were conducted to assess the migration and invasion ability of fibroblasts. The overall survival was analyzed by a Kaplan-Meier analysis. Associations between categorical variables were assessed using the Pearson's Chi-square test or Fisher's exact test.

**Results:** GAC patients with B7-H3 expression showed significantly poorer survival (*P* = 0.012). The overall survival of the group with high B7-H3 expression was significantly worse than the group with low B7-H3 expression in both tumor cells and in stromal cells (*P* = 0.007 and *P* = 0.048, respectively). B7-H3 expression correlated with many clinicopathological data, including tumor stage, tumor depth, lymph node involvement, and survival. Immunofluorescence staining showed that B7-H3 was expressed in tumor cells and α-SMA-positive fibroblasts. Remarkably, high expression of α-SMA was associated with a poor prognosis (*P* = 0.007), and the prognoses of patients with high stromal expression of B7-H3 and α-SMA were significantly worse than that of other combination types (*P* = 0.001). Additionally, the absence of B7-H3 led to decreased secretion of cytokines, such as interleukin (IL)-6 and vascular endothelial growth factor (VEGF), as well as a decline in migration and invasion ability in cancer-associated fibroblasts (CAFs).

**Conclusions:** Patients with high B7-H3 expression either in tumor cells or in stromal cells had significantly poorer overall survival. Stromal B7-H3 expression was mostly detected in α-SMA-positive CAFs. GAC patients with both stromal B7-H3-high and α-SMA-high expression had significantly poorer overall survival, suggesting that stromal B7-H3 and α-SMA expression status can serve as an indicator of poor prognosis for GAC patients.

## Introduction

A gastric adenocarcinoma (GAC) is a malignant tumor that originates from the gastric epithelium (1). Conventional treatments including surgical removal, radiotherapy, and chemotherapy have improved the survival rate of patients to some extent; however, the 5-year survival rate is still low due to the long period of pathogenesis and treatment limitations (2). In recent years, immune checkpoint blockades with monoclonal antibodies against negative costimulatory molecules in tumors have attracted much attention. For instance, cytotoxic T-lymphocyte-associated antigen 4 (CTLA-4) (3, 4), programmed death 1 (PD-1) (5), and programmed death-ligand 1 (PD-L1) (6) have achieved satisfactory therapeutic results in different cancer treatments. Indeed, the initial success of the immune checkpoint PD-1/PD-L1 blockade with monoclonal antibodies has led to more extensive research of other negative costimulatory molecules (7); B7-H3 is an important immune checkpoint member of the B7 and CD28 families, and it is expressed in a wide range of cells including tumor cells, endothelial cells, natural killer cells, B cells, activated macrophages, dendritic cells, monocytes, and fibroblasts (8–12). Notably, B7-H3 is overexpressed in many different tumors, including melanoma and leukemia as well as breast, prostate, ovarian, pancreatic, colorectal, and gastric cancers (13–20). In addition, B7-H3 has been associated with a poor prognosis and poor clinical outcomes of patients.

A previous study has shown that chimeric antigen receptor T cells (CAR-Ts) targeting B7-H3 effectively control tumor growth (21). In addition, anti-B7-H3 monoclonal antibodies were targeted on B7-H3-positive tumor cells to treat patients (22). However, the role of B7-H3 expression on stromal cells has not been established in the tumor microenvironment. The tumor microenvironment, especially immune cell infiltration, appears to be associated with B7-H3 expression. Our laboratory has previously reported the expression and function of B7-H3(+)CD14(+)HLA-DR(–/low) myeloid-derived suppressor cells on human non-small-cell lung cancer (NSCLC) (23). In another study, we analyzed the trend of B7-H3 expression and its relation to CD8 as well as CD68 expression in different stages of gastric carcinogenesis (24). Furthermore, B7-H3 expression in endothelial cells and fibroblasts has been reported (9, 10, 25). CD31, whose expressed is normally restricted to the vascular system, including endothelial cells, platelets, monocytes, and circulating T-cell subsets (26), has several important functions, such as cell migration, angiogenesis, coagulation, maintenance of vascular permeability, and regulation of apoptosis (27, 28). However, another important cell type in the tumor microenvironment, activated cancer-associated fibroblasts (CAFs), may play a vital role in tumor development and is a potential target for cancer treatment (29). As major components of the tumor stroma, CAFs promote tumor growth by secreting various cytokines (30). It has been shown that there are different CAF populations secreting distinct profiles of cytokines in different cancers (31, 32). CAFs produce the ultrastructure of alpha-smooth muscle actin (α-SMA) (30). Studies have demonstrated that the α-SMA expression correlates with the activation of myofibroblasts (33). Furthermore, α-SMA expression is significantly associated with the progression of many different malignant tumors, such as lung adenocarcinoma, osteosarcomas, and head and neck squamous cell carcinoma (34–36). Nevertheless, the relationships between B7-H3 expression in fibroblasts with the patient prognosis as well as with other clinicopathological data remain to be elucidated.

In the present study, we performed an immunohistochemical analysis of B7-H3, CD31, and α-SMA protein expression in 268 GAC samples using a tissue microarray (TMA) in order to assess the importance of B7-H3 expression in tumor and stromal cells. We analyzed the correlation between B7-H3 expression and CD31 or α-SMA expression as well as the impact of B7-H3 expression combined with CD31 or SMA expression on patient survival. Finally, we analyzed the function of B7-H3 expression in fibroblasts, which may lead to a poor patient prognosis.

## Materials and Methods

### Patients and Specimens

This retrospective study included 268 samples from GAC patients (210 males and 58 females) aged between 33 and 88 years old (average age: 64.5 ± 10.8 years old). Thirty-six patients with stage IV were enrolled, and four of these patients underwent gastrectomy with chemotherapy. These patients were diagnosed with GAC during 2011 by two qualified pathologists from the Department of Pathology, The First Affiliated Hospital, Soochow University, Suzhou, China. The clinical features of 268 GAC patients are shown in Table S1. All samples were acquired from surgical resection, fixed in neutral buffered formalin, and embedded in paraffin. At the same time, the clinical and pathological data of each patient, including gender, age, differentiation, tumor stage, tumor depth, lymph node metastasis, and survival, were retrieved. Overall survival was defined as the time from the start of treatment to the date of death. It needs to be emphasized that only 198 patients had information on overall survival. This study was approved by Clinical Research Ethics Committee of The First Affiliated Hospital of Soochow University (approval number: 2018121).

### Tissue Microarray (TMA) Construction

An experienced pathologist marked the representative points in 268 paraffin-embedded specimens based on the hematoxylin-eosin-stained slides. The TMAs were constructed using an automated tissue array instrument (Beecher ATA-27; Estigen OU, Tartu, Estonia). Two tissue cores (1 mm), including tumor cell-based and stromal cell-based tissues, were taken from each paraffin-embedded specimen. The tissue cores were placed in the TMA blocks according to prearranged modules. Then the completed TMA blocks were cut into 4 μm sections by an experienced pathologist.

### Immunohistochemistry

Immunohistochemical staining was performed according to a protocol described previously (24). Incubation with mouse antibodies against human CD31, α-SMA, CD19, CD11c, or CD68 (Ready to use; ZSGB-BIO, China); mouse anti-human Pan cytokeratin (PanCK) antibody (ab86734 1:50; Abcam, USA); or goat anti-human B7-H3 polyclonal antibody (R&D Systems, Cat# AF1027, RRID:AB_354546) was performed at 4°C overnight, and this was followed by incubation with horseradish peroxidase (HRP)-conjugated secondary antibody (GP016029 for anti-goat or GP016129 for anti-mouse and rabbit, Genetech, China).

### Assessment of Tissue Staining

All immunohistochemical slides were independently and blindly reviewed by two pathologists and scanned with a Dmetrix imaging system (Dmetrix, Tucson, AZ, USA). The slides were screened at low power magnification (100×) for any staining. The staining intensities were determined under a higher magnification (400×). Every patient tissue core of either intratumoral or peritumoral areas was reviewed and scored. The percentage of positively stained cells was calculated: the average number of positive cells/the total number of cells from all fields ×100. The staining histoscore of the slides was classified as 0–3 (0, no staining; 1, <10% staining; 2, 10–50% staining; and 3, >50% staining). The sections scored as 0 and 1 were defined as the low-expression group, and sections scored as 2 and 3 were defined as the high-expression group. The histoscore of staining quantification was assessed according to the formula (3 + percent cells) × 3 + (2 + percent cells) × 2 + (1 + percent cells) × 1. This yielded an immunohistochemical score ranging from 0 to 300 (37).

### Double Immunofluorescence

After slides with goat anti-human B7-H3 polyclonal antibody were incubated with HRP-conjugated secondary antibody, as described in the immunohistochemical analysis section, the tyramide signal amplification (TSA) Fluorescence reagent (#2384212, Cyanine 3 System, PerkinElmer, USA) was added, and the reaction proceeded for 10 min at room temperature. The slides were then washed and treated with antigen retrieval reagents again. After blocking, the slides were incubated with the other antibody against CD31, α-SMA, or PanCK for 1 h at 37°C. The slides were then incubated with the TSA fluorescence reagent (#2336651, Fluorescein System, PerkinElmer, USA) after incubation with HRP-conjugated secondary antibody. Finally, DAPI (Dojindo Laboratories, Kumamoto, Japan) was used for counterstaining the nuclei, and images were obtained by a fluorescence microscope (Nikon Eclipse Ni, Tokyo, Japan) or a laser scanning confocal microscope (Olympus IX83, Tokyo, Japan).

### Isolation of Fibroblasts and Immunocytochemistry

Normal fibroblasts (NFs) and CAFs were isolated, as described previously (38, 39). The CAFs were isolated from GAC samples obtained from surgery, and the NFs were isolated from their matched non-malignant adjacent tissues, taken at least 10 cm from the outer tumor margin. The samples were rinsed and soaked with 10× double-antibiotic with amphotericin B, and the minced tissue was then digested with collagenase type I, collagenase type III, and hyaluronidase (1.5 mg/mL, Sigma-Aldrich, St. Louis, MO, USA) at 37°C with agitation for 2–3 h in Dulbecco's modified Eagle medium (DMEM) containing 10% fetal bovine serum (FBS). The digested cell solution was centrifuged and plated, and cells of passages 3–7 were selected for the next experiment. The purity of CAFs and NFs was determined by analyzing the fibroblast-specific protein vimentin and the activated fibroblast marker fibroblast activation protein (FAP-α). CAFs and NFs for immunofluorescence analysis were fixed with 4% formaldehyde for 15 min and washed with phosphate-buffered saline (PBS) three times. The cells were then permeabilized and blocked in PBS with 0.1% Triton-X100 followed by 2% FBS for 1 h at room temperature. After blocking, the samples were incubated with primary antibodies including rabbit anti-human vimentin (CST, Cat# 5741s, RRID:AB_10695459), mouse anti-human CD31 (Cat# ZM-0044, ZSGB-BIO, China), goat-anti-human B7-H3 (R&D Systems, Cat# AF1027, RRID:AB_354546), sheep-anti-human FAP-α (R&D Systems, Cat# AF3715, RRID:AB_2102369), and mouse anti-human α-SMA (Cat# ZM-0003, ZSGB-BIO, China) overnight at 4°C. Moreover, incubation with HRP-conjugated secondary antibodies (Life Technologies, Duren, DE, USA) and the TSA System (#2384212, Cyanine 3 System, PerkinElmer, MA, USA) were separately carried out for 30 min and 10 min, respectively, at room temperature. Finally, DAPI (Dojindo Laboratories, Kumamoto, Japan) was then used for counterstaining of nuclei, and images were obtained using fluorescent microscopy (Nikon Eclipse Ni, Tokyo, Japan) or laser scanning confocal microscopy (Olympus IX83, Tokyo, Japan).

### Small Interfering RNA (siRNA)

The siRNA targeting specific human B7-H3 sites (si996 and si1041) and the negative control siRNA (siNC) were synthesized by GenePharma (Shanghai, China). The sequences of 996 and 1041 siRNA were 5′-GCUGUCUGUCUGUCUCAUUTT-3′ and 5′-GUGCUGGAGAAAGAUCAAATT-3′, respectively.

### Enzyme-Linked Immunosorbent Assay (ELISA)

Primary fibroblasts were cultured in DMEM containing 10% human serum until they reached 80% confluency. Next, the cells were cultured in fresh serum-free media for 24 h, the supernatant was collected, and cytokines (IL-6 and VEGF) were measured in duplicate using specific ELISA kits purchased from Thermo Fisher (Waltham, MA, USA). All experiments were performed according to the manufacturer's instructions.

### Quantitative Reverse Transcription–Polymerase Chain Reaction (qRT-PCR)

Isolation of total RNA was carried out using NucleoSpin RNAII (#740955, Macherey-Nagel, Duren, Germany), and the first-strand cDNA was then generated using the PrimeScript RT reagent kit (#RR037A, TaKaRa, Dalian, China). The qRT-PCR was performed using a SYBR Premix Ex Taq II kit (#RR820L, TaKaRa), according to the manufacturer's instructions. The primer sequences are listed in Table S2. Data were collected and analyzed using a CFX Connect Real-Time PCR Detection System instrument (Bio-Rad, Hercules, CA, USA).

### Statistical Analysis

Statistical analysis was performed using SPSS software (v19.0; IBM Corporation, Armonk, NY, USA). A comparison between groups in terms of B7-H3, CD31, or α-SMA protein expression was performed using the chi-squared test. The correlation of B7-H3 with CD31 and α-SMA protein expression was analyzed using Pearson's correlation. A Kaplan-Meier analysis was applied for the survival analysis. Differences between curves were estimated by the two-sided log-rank test. *P*-values < 0.05 were defined as statistically significant.

## Results

**Figure 1:**
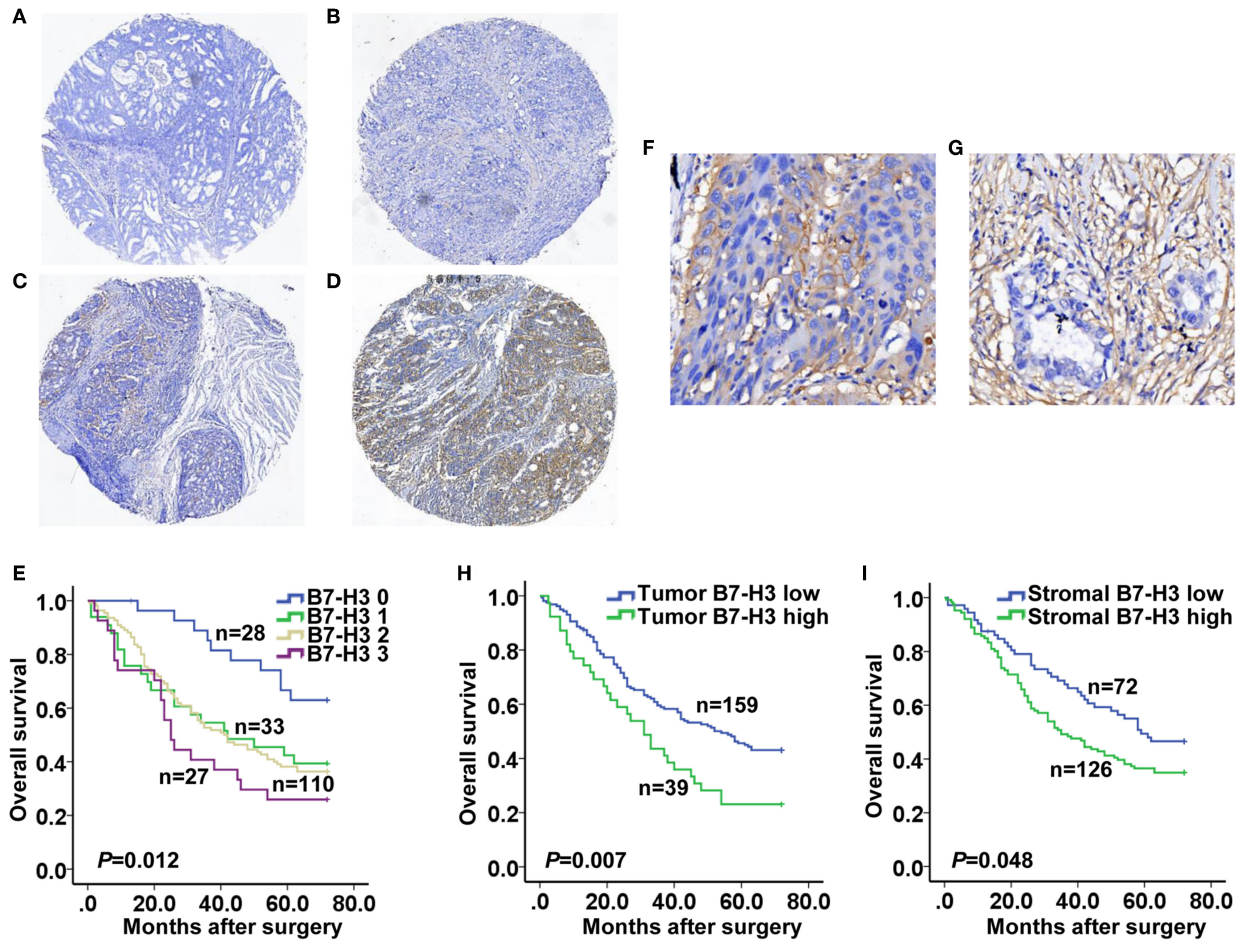
B7-H3 expression and its relationship with overall survival of gastric adenocarcinoma patients. **(A–D)** Immunohistochemical characteristics of different intensity scores for B7-H3. **(E)** Kaplan-Meier curves according to B7-H3 different intensity score. **(F)** B7-H3 positive staining in tumor cells. **(G)** B7-H3 positive staining in stromal cells. **(H,I)** Kaplan-Meier curves according to low and high expression of B7-H3 (B7-H3^low^ and B7-H3^High^) in tumor or stromal cells.

In this study, we examined 268 tumors taken from patients diagnosed with GAC using immunohistochemistry and TMAs. B7-H3 expression was scored as 0, 1, 2, and 3 according to the immunohistochemical expression intensity (Figures 1A–D). The results showed that B7-H3 expression was significantly correlated with the tumor stage (*P* = 0.000), tumor depth (*P* = 0.001), lymph node involvement (*P* = 0.020), and survival (*P* = 0.003) (Table 1). Patients with a higher expression of B7-H3 had a significantly poorer overall survival (*P* = 0.012) (Figure 1E). We also found that B7-H3 was expressed in both tumor and stromal cells (Figures 1F,G). To further evaluate the clinical and pathological significance of B7-H3 expression on tumor or stromal cells in GAC, we statistically analyzed the correlation between B7-H3 expression in tumor or stromal cells and the clinicopathological data. In our study, 50 (18.7%) patients had high B7-H3 expression in tumor cells, while 168 (62.7%) had high expression in stromal cells (Table 1). When B7-H3 expression in tumor cells (tumor B7-H3) was correlated with the clinicopathological data, we found higher tumor volume (*P* = 0.023), tumor stage (*P* = 0.002), tumor depth (*P* = 0.001), and lymph node involvement (*P* = 0.006) as well as a lower survival (*P* = 0.020) in the group with high expression of tumor B7-H3 (Table 1). High expression of B7-H3 in tumors was significantly correlated with a poorer overall survival (*P* = 0.007) (Figure 1H). When B7-H3 expression in stromal cells (stromal B7-H3) and the clinicopathological data were correlated, the tumor stage (*P* = 0.009) and tumor depth (*P* = 0.009) were significantly positively associated with B7-H3 expression (Table 1). High expression of B7-H3 in stromal cells was also correlated with a poorer overall survival (*P* = 0.048) (Figure 1I).

**Table 1:** B7-H3 expression and clinical features in 268 gastric adenocarcinoma patient samples.

**Clinical or pathological features**	**Total No**.	**B7-H3 expression**				***P*-value**	**Tumor cells**^**a**^		***P*-value**	**Stromal cell**^**b**^		***P*-value**
		**0**	**1**	**2**	**3**		**Low^**c**^**	**High^**d**^**		**Low^**c**^**	**High^**d**^**	
All cases	268	38	46	151	33		218	50		100	168	
Age						0.723			0.609			0.180
<70	164	24	23	98	19		135	29		56	108	
≥70	104	14	23	53	14		83	21		44	60	
Sex						0.641			0.285			0.678
Male	58	6	13	36	3		50	8		23	35	
Female	210	32	33	115	30		168	42		77	133	
Tumor volume (cm^3^)						0.087			0.023			0.478
<5	186	35	29	96	26		158	28		72	114	
≥5	82	3	17	55	7		60	22		28	54	
Tumor differentiation						0.066			0.733			0.077
Well	6	2	0	4	0		5	1		3	3	
Moderate	121	14	16	69	22		97	24		36	85	
Poor	141	22	30	78	11		116	25		61	80	
Tumor stage						0.000			0.002			0.009
0	11	5	3	3	0		11	0		8	3	
I	32	6	7	16	3		30	2		14	18	
II	66	12	13	35	6		55	11		28	38	
III	123	13	16	75	19		97	26		38	85	
IV	36	2	7	22	5		25	11		12	24	
Tumor depth						0.001			0.001			0.009
T1	36	9	9	16	2		34	2		19	17	
T2	34	7	9	16	2		33	1		16	18	
T3	169	19	26	98	26		130	39		57	112	
T4	29	3	2	21	3		21	8		8	21	
LN involvement						0.020			0.006			0.170
N0	85	18	16	43	8		74	11		38	47	
N1	62	10	9	35	8		55	7		22	40	
N2	56	7	6	32	11		42	14		17	39	
N3	65	3	15	41	6		47	18		23	42	
Metastasis						0.269			0.091			0.634
M0	238	37	39	133	29		197	41		90	148	
M1	30	1	7	18	4		21	9		10	20	
Death						0.003			0.020			0.089
–	78	18	13	40	7		69	9		34	44	
+	120	10	20	70	20		90	30		38	82	

a *B7-H3 expression on tumor cells*.

b *B7-H3 expression on stromal cells*.

c *B7-H3 low expression*.

d *B7-H3 high expression*.

**Figure 2:**
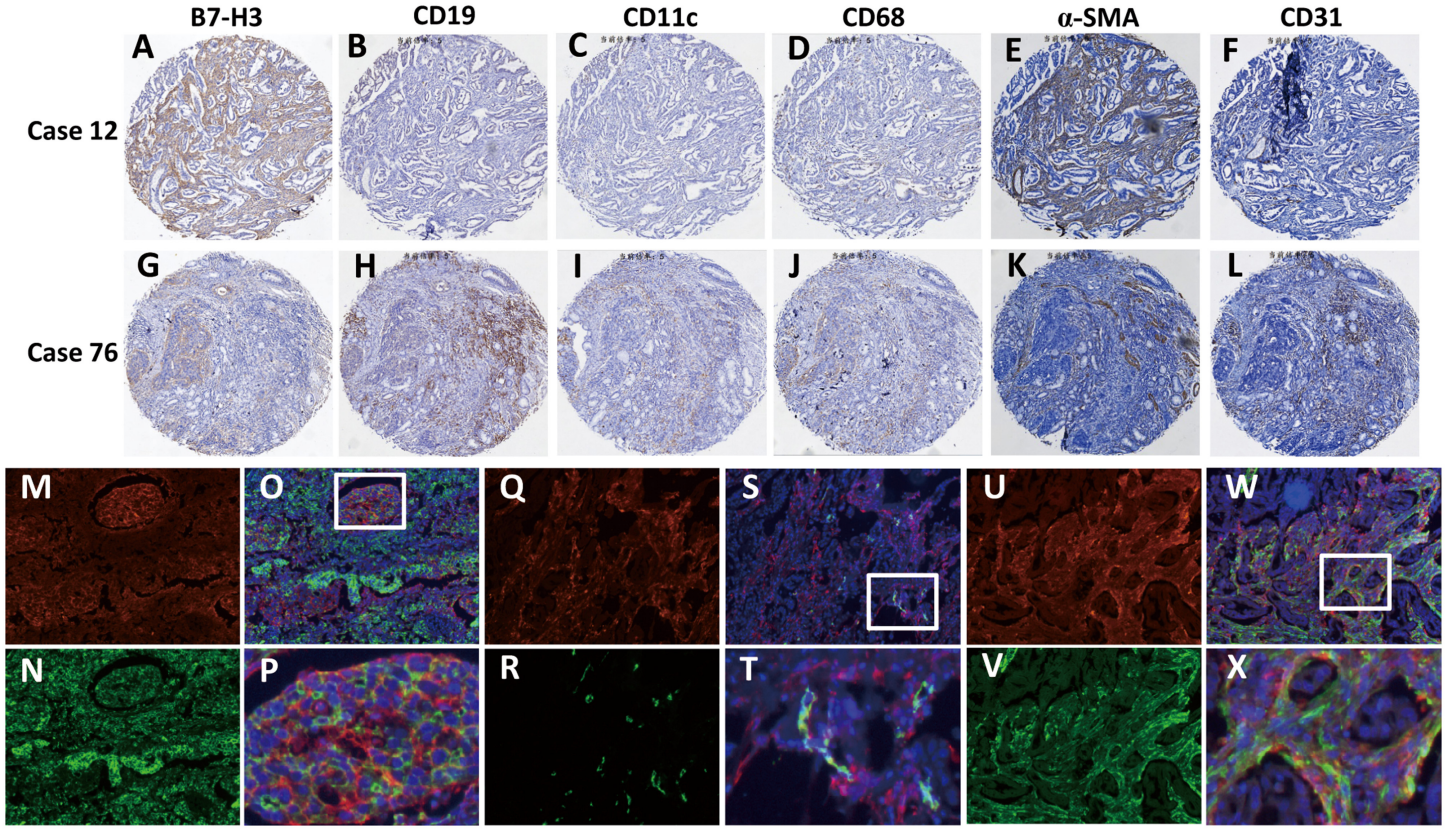
Identification of B7-H3-positive cells in gastric adenocarcinoma tissues. **(A–F)** A tumor with high B7-H3 expression in stromal cells and its CD19, CD11c, CD68, CD31, and α-SMA expression status. **(G–L)** A tumor with high B7-H3 expression in tumor cells and its CD19, CD11c, CD68, CD31, and α-SMA expression status. **(M–X)** Double immunofluorescence analysis of B7-H3 and PanCK, CD31, or α-SMA in gastric adenocarcinoma tissues. DAPI fluorescence is shown in blue, B7-H3 immunofluorescence is shown in red, and PanCK, CD31, or α-SMA immunofluorescence is shown in green; **(M,N)** B7-H3 and PanCK immunofluorescent staining; **(O)** the merge picture of B7-H3 and PanCK with DAPI immunofluorescent staining (×100 magnification); **(P)** magnification of the dotted line frames of **(O) (**×400 magnification**)**; **(Q,R)** B7-H3 and CD31 immunofluorescent staining; **(S)** the merge picture of B7-H3 and CD31 with DAPI immunofluorescent staining (×100 magnification); **(T)** magnification of the dotted line frames of **(S) (**×400 magnification**)**; **(U,V)** B7-H3 and α-SMA immunofluorescent staining; **(W)** the merge picture of B7-H3 andα-SMA with DAPI immunofluorescent staining (×100 magnification); **(X)** magnification of the dotted line frames of **(W) (**×400 magnification**)**.

To further explore the correlation between B7-H3 expression in different types of interstitial cells and the patient's pathological data and survival, we analyzed the cellular localization of B7-H3 expression in gastric cancer tissues. Since it has been reported that B7-H3 is expressed in tumor cells as well as in various immune cells, including endothelial cells, natural killer cells, B cells, macrophages, dendritic cells, and fibroblasts (8–12), we examined the expression of B7-H3 in stromal (case 12) and tumor (case 76) cells together with the expression of the immune cell markers CD19, CD11c, CD68, CD31, and α-SMA in serial sections (Figures 2A–L). The analysis showed that B7-H3 expression in stromal cells was in accordance with α-SMA expression (Figures 2A,E), while the B7-H3-positive tumor cells presented with different immune cell infiltration (Figures 2G–L). According to a crosstab rank correlation test, no significant correlation was found between B7-H3 and CD19 (*r* = 0.034, *P* = 0.581) or CD11c (*r* = 0.043, *P* = 0.480) expression. However, there was a significant correlation between B7-H3 and CD68 (*r* = 0.146, *P* = 0.016), CD31(*r* = 0.218, *P* = 0.000), or α-SMA (*r* = 0.206, *P* = 0.001) expression (Figure S1). Among them, the correlation between B7-H3 and CD31 or α-SMA expression was much higher than that of B7-H3 and CD68 expression.

Based on these analyses, we further used immunofluorescence staining to confirm B7-H3-expressing cells. The localized expression of B7-H3 and PanCK showed that only some of the tumor cells expressed B7-H3; however, some that were PanCK-positive did not express B7-H3 (Figures 2M–P). Next, we examined the coexpression of B7-H3 with the endothelial cell marker CD31 and the CAFs marker α-SMA in stromal cells. The results showed that B7-H3 and CD31 were co-expressed less frequently (Figures 2Q–T), and the vast majority of the stromal cells expressing B7-H3 did not express CD31, suggesting that B7-H3 and CD31 expression had no apparent consistency. Nevertheless, unlike CD31, α-SMA expression was significantly consistent with B7-H3 expression in fibroblasts, although their expression did not have significant colocalization (Figures 2U–X).

**Figure 3:**
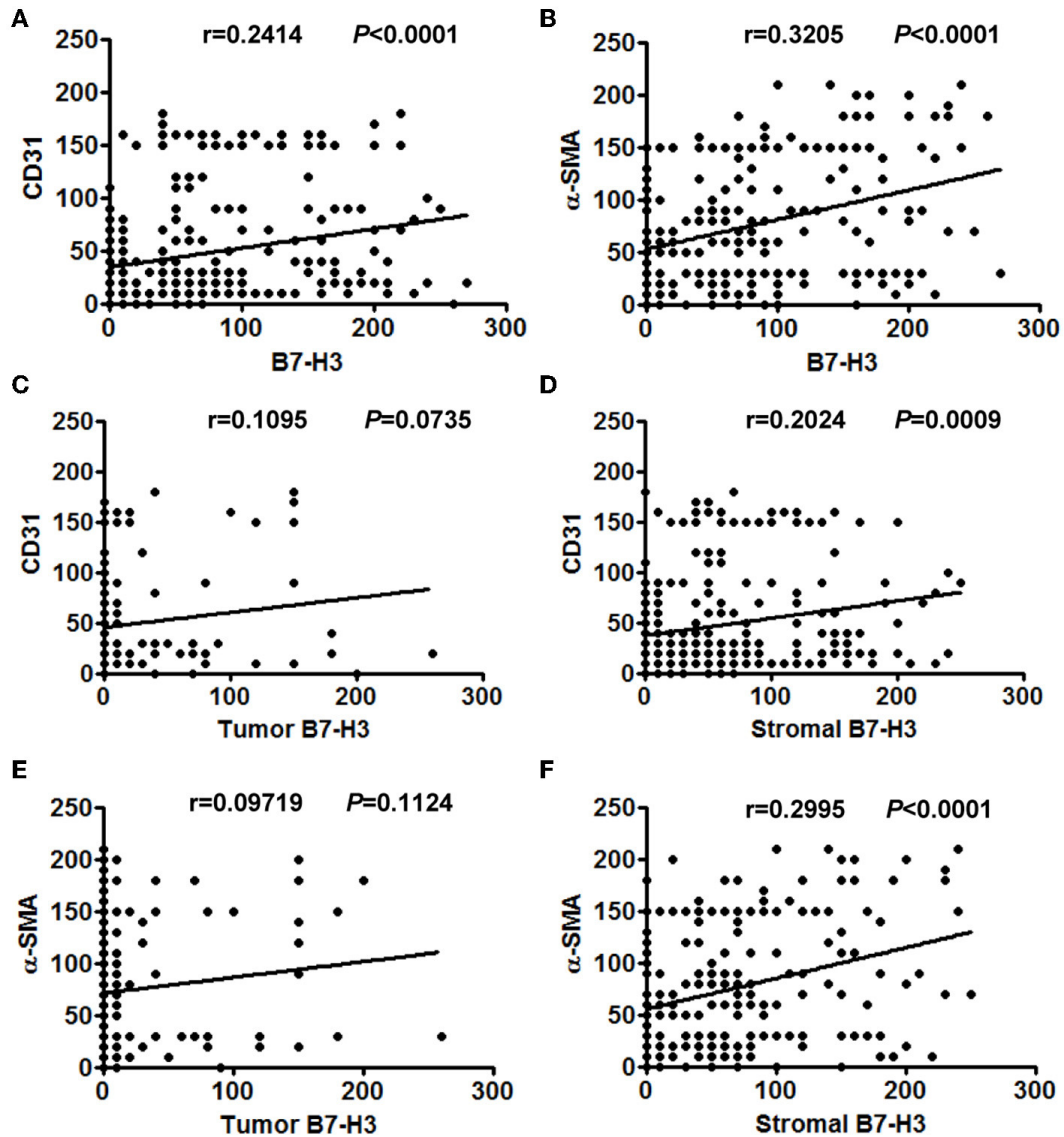
The correlation between different expression levels of B7-H3 and CD31 or α-SMA expression. **(A)** Total B7-H3 expression and CD31. **(B)** Total B7-H3 expression and α-SMA. **(C)** Tumor B7-H3 expression and CD31. **(D)** Stroma B7-H3 expression and CD31. **(E)** Tumor B7-H3 expression and α-SMA. **(F)** Stroma B7-H3 expression and α-SMA.

Considering that infiltrated endothelial cells and fibroblasts were associated with B7-H3 expression, we further evaluated the clinical and pathological significance of CD31 and α-SMA expression. The results showed that CD31 expression was related to the degree of tumor differentiation (*P* = 0.029), i.e., low CD31 expression was associated with a lower degree of tumor differentiation (Table 2). The α-SMA expression was significantly positively correlated with the tumor stage (*P* = 0.023), metastasis (*P* = 0.027), and poor survival (*P* = 0.000) (Table 2). We then analyzed the correlation between total B7-H3 expression and CD31 or α-SMA expression. A positive correlation was observed between B7-H3 and CD31 (*r* = 0.2414, *P* < 0.0001), and B7-H3 and α-SMA (*r* = 0.3205, *P* < 0.0001) (Figures 3A,B). While B7-H3 expression in tumors was considered, no significant correlation with CD31 or α-SMA expression was observed (*r* = 0.1095, *P* = 0.0735; *r* = 0.09719, *P* = 0.1124) (Figures 3C,E). However, stromal B7-H3 expression was positively correlated with CD31 or α-SMA expression (*r* = 0.2024, *P* = 0.0009; *r* = 0.2995, *P* < 0.0001) (Figures 3D,F).

**Table 2:** CD31 and α-SMA expression and clinical features in 268 gastric adenocarcinoma patient samples.

**Clinical or pathological features**	**Total No**.	**CD31**				***P*-value**	**α-SMA**				***P*-value**
		**0**	**1**	**2**	**3**		**0**	**1**	**2**	**3**	
All cases	268	24	136	73	35		11	88	108	61	
Age						0.859					0.227
<70	164	17	79	45	23		9	53	69	33	
≥70	104	7	57	28	12		2	35	39	28	
Sex						0.755					0.521
Female	58	6	31	12	9		2	16	27	13	
Male	210	18	105	61	26		9	72	81	48	
Tumor volume (cm^3^)						0.800					0.111
<5	186	14	100	49	23		8	61	84	33	
≥5	82	10	36	24	12		3	27	24	28	
Tumor differentiation						0.029					0.820
Well	6	0	5	1	0		1	2	3	0	
Moderate	121	14	62	35	10		3	45	40	33	
Poor	141	10	39	37	25		7	41	65	28	
Tumor stage						0.100					0.023
0	11	2	7	2	0		1	2	7	1	
1	32	3	18	9	2		3	11	13	5	
2	66	5	34	15	12		3	22	26	15	
3	123	10	64	33	16		4	46	48	25	
4	36	4	14	13	5		0	7	14	15	
Tumor depth						0.107					0.094
T1	36	4	23	6	3		2	8	22	4	
T2	34	3	16	11	4		3	13	11	7	
T3	169	15	84	46	24		6	62	61	40	
T4	29	2	13	10	4		0	5	14	10	
LN involvement						0.182					0.138
N0	85	7	48	21	9		6	28	33	18	
N1	62	8	31	12	11		4	18	31	9	
N2	56	4	28	20	4		1	17	22	16	
N3	65	5	29	20	11		0	25	22	18	
Metastasis						0.533					0.027
M0	238	21	124	62	31		11	81	96	50	
M1	30	3	12	11	4		0	7	12	11	
Death						0.956					0.000
–	78	6	43	20	9		6	35	29	8	
+	120	11	60	37	12		3	36	43	38	

**Figure 4:**
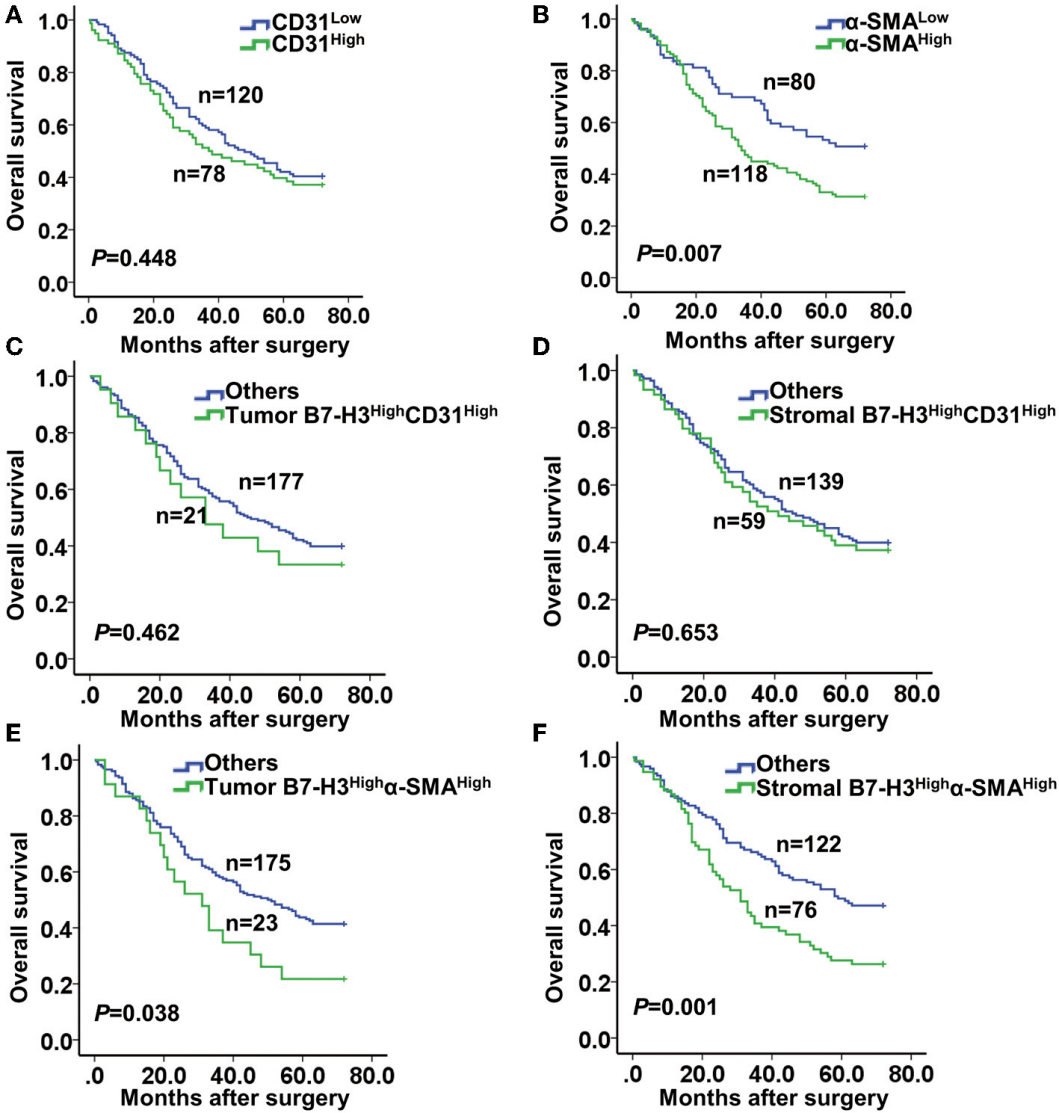
Kaplan-Meier curve of survival according to the expression of CD31, α-SMA, and B7-H3. **(A)** Kaplan-Meier curves according to low and high expression of CD31 (CD31^low^ and CD31^High^). **(B)** Kaplan-Meier curves according to low and high expression of α-SMA (α-SMA^low^ and α-SMA^High^). **(C,D)** Kaplan-Meier curves according to the combination of expression of B7-H3 and CD31: **(C)** B7-H3^High^ CD31^High^ vs. others (including B7-H3^High^ CD31^Low^, B7-H3^Low^ CD31^High^, and B7-H3^Low^ CD31^Low^) in tumor cells; **(D)** B7-H3^High^ CD31^High^ vs. others in stromal cells. **(E,F)** Kaplan-Meier curves according to the combination of expression of B7-H3 and α-SMA: **(E)** B7-H3^High^ α-SMA^High^ vs. others (including B7-H3^High^ α-SMA^Low^, B7-H3^Low^ α-SMA^High^ and B7-H3^Low^ α-SMA^Low^) in tumor cells; **(F)** B7-H3^High^ α-SMA^High^ vs. others in stromal cells.

Next, we performed a Kaplan-Meier survival analysis of patients according to CD31 or α-SMA expression. The results showed no significant correlation between the high and the low CD31 expression groups (*P* = 0.448) (Figure 4A). However, patients with high α-SMA expression had a significantly poorer overall survival compared with patients with low α-SMA expression (*P* = 0.007) (Figure 4B). Furthermore, we analyzed the Kaplan–Meier survival curve of patients in relation to combined B7-H3 and CD31 or α-SMA expression. Whether B7-H3 was expressed in tumor or stromal cells, no significant difference in the overall survival rate was observed between the B7-H3^High^CD31^High^ and the others (*P* = 0.462 and *P* = 0.653, respectively) (Figures 4C,D). However, the survival curve analysis of patients with tumor B7-H3^High^α-SMA^High^ expression showed a significantly reduced survival rate compared to the others (*P* = 0.038) (Figure 4E). Remarkably, the survival of patients with stromal B7-H3^High^α-SMA^High^ expression was more significantly decreased compared to the survival of the others (*P* = 0.001) (Figure 4F).

**Figure 5:**
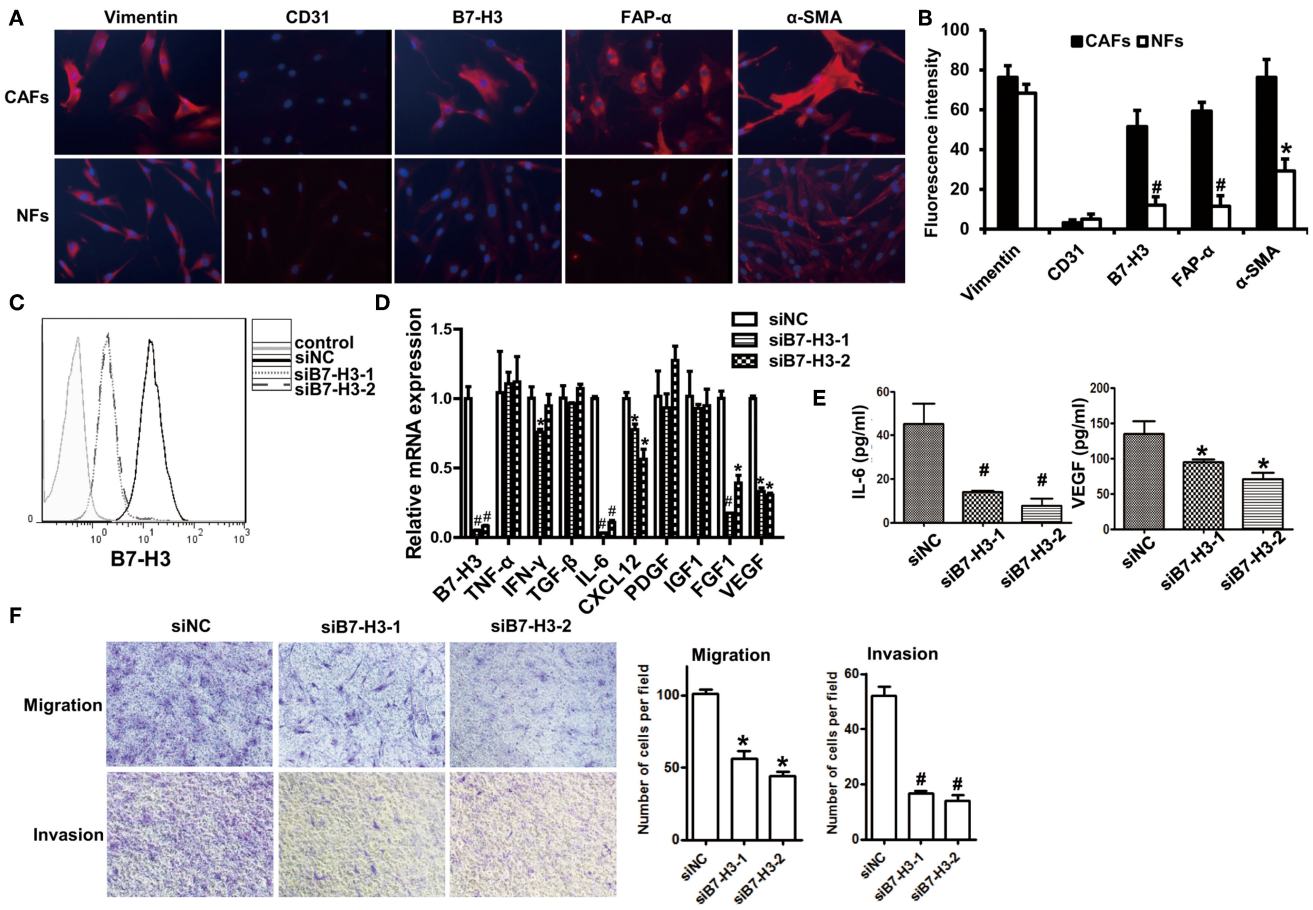
Interfering B7-H3 expression in CAFs-blocked cytokine secretion and decreased migration and invasion ability. **(A)** immunofluorescence images of vimentin, CD31, B7-H3, FAP-α, and α-SMA in CAFs and NFs. DAPI fluorescence is shown as blue. Vimentin, CD31, B7-H3, FAP-α, and α-SMA immunofluorescence is shown as red. **(B)** The Fluorescence intensity statistical results of vimentin, CD31, B7-H3, FAP-α, and α-SMA. Mean ± SEM, *P < 0.05 and ^#^*P* < 0.01 by Student's *t*-test. **(C)** B7-H3 protein expression in fibroblasts after treatment with B7-H3 siRNA. **(D)** The relative mRNA expression levels of B7-H3 and cytokines are indicated after treatment with B7-H3 siRNA. Mean ± SEM, *P < 0.05 and ^#^*P* < 0.01 by Student's *t*-test. **(E)** The IL-6 and VEGF expression levels as detected by ELISA. Mean ± SEM, *P < 0.05 and ^#^*P* < 0.01 by Student's *t-*test. **(F)** The transwell tests were conducted to assess the migration and invasion ability of fibroblasts.

To further investigate the function of B7-H3 expression in CAFs, we isolated CAFs from tumor tissue and NFs from normal gastric tissue. We examined B7-H3, α-SMA, FAP-α, vimentin, and CD31 expression in order to identify the activation of fibroblasts and to further explore the biological functions of B7-H3-expressing CAFs. The immunofluorescence results showed that vimentin expression was positive and CD31 was negative in CAFs and NFs, which meant that fibroblasts were successfully isolated. However, α-SMA and FAP-α, which, along with B7-H3, are widely used as CAFs markers, were more highly expressed in CAFs than in NFs (Figures 5A,B). To confirm the possible function of B7-H3 expression in CAFs leading to a poor prognosis in patients, we treated CAFs with B7-H3 siRNA (Figure 5C) and performed cytokine detection. We found a significant decrease in mRNA level of several cytokines, including IL-6, CXCL12, FGF1, and VEGF (Figure 5D), as well as in the protein level of IL-6 and VEGF (Figure 5E). Finally, after B7-H3 depletion, we found that the migration and invasion ability of CAFs was significantly inhibited (Figure 5F).

## Discussion

The aim of this study was to examine the status of B7-H3 in GAC tumor and stromal cells as well as its relation to α-SMA expression. Our results showed that B7-H3 was expressed in both tumor cells and stromal cells. Stromal B7-H3 expression was mostly detected in α-SMA-positive CAFs. Tumor and stromal B7-H3 expression were positively correlated with α-SMA expression, and the combination of high B7-H3 and high α-SMA expression could be used as an indicator of a poor prognosis for GAC. In addition, our results showed that the secretion of multiple cytokines, including IL-6 and VEGF, and the ability of migration and invasion were significantly decreased in CAFs, which were treated with B7-H3 siRNA. These results also suggest that both B7-H3 and fibroblasts may serve as targets for the treatment of GAC.

It has been reported that B7-H3 is overexpressed in gastric cancer and that it correlates with a poor outcome in gastric cancer patients. In addition, the absence of B7-H3 expression decreased gastric cancer cell migration and invasion as well as reduced tumor metastasis in an orthotropic transplantation gastric cancer model (40). Li et al. also found that shRNA-mediated B7-H3 silencing in the N87 gastric cancer cell line suppressed cell migration and invasion *in vitro* and *in vivo*, and knockdown of B7-H3 resulted in a longer survival time (16). Besides, B7-H3 appears to be a useful blood marker for predicting tumor progression in gastric cancer (41). Consistent with these findings, our study indicated that patients with a higher expression of B7-H3 had significantly poorer overall survival. The expression of B7-H3 in tumor/parenchymal cells was more strongly correlated with CD8-positive and CD68-positive cell infiltration and was markedly enhanced at the stages of neoplasia and GAC (24). The function of B7-H3 in promoting gastric cancer development has been studied in depth. However, the function of B7-H3 in stromal cells needs to be further elaborated.

It has been showed that B7-H3 is positively expressed in the cell membrane and cytoplasm of gastric cancer and adenoma cells (42). However, we found that B7-H3 was mostly expressed in the cell membrane and rarely expressed in the cytoplasm. No expression of B7-H3 was observed in the nucleus. Several prior reports have established that B7-H3 expression is present in endothelial cells, activated macrophages, dendritic cells, B cells, natural killer cells, etc. (8–12). In agreement with these studies, we also detected the expression of B7-H3 in these cell types, including CD31-positive endothelial cells, α-SMA-positive fibroblasts, CD11c-positive dendritic cells, CD68-positive macrophages, and CD19-positive B cells (Figure 2). However, in our study, the infiltration of B7-H3-positive stromal cells was inconsistent with these cells. Via a correlation analysis, we found that the expression of B7-H3 was not significantly correlated to CD19 or CD11c expression, suggesting that B7-H3 in these two types of cells may not play the main role in the microenvironment. In addition, we found that there was a significant correlation between B7-H3 and CD68 expression. This result is consistent with our previous findings (24), and our group also found that B7-H3 regulates the differentiation of tumor-associated macrophages in human colorectal carcinoma (43). However, the correlation between B7-H3 and CD31 or α-SMA expression was much higher than that of B7-H3 and CD68 expression, suggesting that B7-H3 may play a vital role in the tumor microenvironment, including endothelial cells and fibroblasts. Several studies have shown that B7-H3 is expressed in a variety of cancer-associated endothelial cells, regulates T-cell activity, and is directly related to patient prognosis (9, 44–46). We further analyzed and found that B7-H3 expression was consistent with the infiltration of CD31-positve endothelial cells and α-SMA-positive fibroblasts and was even more consistent with the infiltration areas of fibroblasts (Figure 2). Is the function of B7-H3 expression in fibroblasts the main function of B7-H3 in the tumor microenvironment? The survival curves of B7-H3 in combination with CD31 and α-SMA in our study further confirm this idea. In the analysis of patients' prognosis, our results were in accordance with those of Sun et al. (47) and Ingebrigtsen et al. (10), and no significant difference in the overall survival of patients with B7-H3-expressing endothelial cells relative to other patients was observed. Meanwhile, the prognosis of patients with high B7-H3 expression in tumor or stromal cells, especially high B7-H3 expression in stromal cells and high α-SMA expression, was significantly worse than that of other combination types. This result indicates that the expression of B7-H3 in fibroblasts may play an important role in tumor progression. However, the relationship between B7-H3 and fibroblasts has not been extensively studied.

In tumor tissues, fibroblasts interact with tumor cells to promote tumor growth and metastasis as well as tumor proliferation, invasion, and metastasis (48). Negative costimulatory molecules, as important molecules of tumor immune escape, are associated with fibroblasts. It has been reported that antibodies targeting fibroblast-derived CXCL12 and PD-L1 can synergistically treat pancreatic cancer (49). Furthermore, PD-L1 and PD-L2 expressed in colon fibroblasts can regulate the activity of CD4^+^ T cells (50). A recent study also has revealed that PD-L1 and PD-L2 colocalize with CD90-positive and vimentin-positive fibroblasts in the mouse epidermis (51). Additionally, B7-H3 is highly expressed in fibroblast-like synoviocytes, where it regulates T-cell function (52). Therefore, the role of B7-H3 expression in fibroblasts associated with tumor cells and immune cells deserves further study.

It is known that myofibroblasts and unactivated fibroblasts are present in tumor tissues. Myofibroblasts in tumors are differentiated from fibroblasts stimulated by tumor cell damage (29, 30, 53). They are the main CAFs, which highly express α-SMA. Moreover, recent studies have revealed that, instead of tumor cells, CAFs contribute to tumor proliferation, invasion, and metastasis via secretion of various growth factors, cytokines, chemokines, and degradation of extracellular matrix proteins (54). IL-6 produced by activated fibroblasts induces tumor angiogenesis (55) and promotes chemoresistance via CXCR7 in esophageal squamous cell carcinoma (56). Activated CAFs further promote cancer progression via secreting angiogenic cytokines including VEGF, matrix metalloproteinase (MMP)-2, MMP9, basic fibroblast growth factor and transforming growth factor-β (57). Moreover, different subgroups of CAFs may play various roles in tumors. CD10^+^GPR77^+^ CAFs can promote tumor stemness by activating nuclear factor-κB signaling to produce IL-6 and IL-8 (58). In our experiments, the group with high stromal B7-H3 expression and high α-SMA expression had a significantly poorer prognosis compared with the other groups combined. Stromal B7-H3 was not only related to the migration and invasion ability of CAFs, but it also was associated with the secretion of various cytokines. From these findings, we speculate that the infiltration of B7-H3-positive CAFs may serve as a promising clinical biomarker to predict the prognosis for GAC patients. Although a B7-H3 receptor has not been found, B7-H3 can regulate the survival, proliferation, and glycolysis of tumor cells through various signaling pathways, including hypoxia-inducible factor-1α, nuclear factor-κB, Stat3, and phosphoinositide 3-kinase (59). In addition, our recent study has found that B7-H3 promotes aerobic glycolysis and chemoresistance in colorectal cancer cells by regulating hexokinase 2 (60). Therefore, further research on the function and molecular mechanism of B7-H3 in fibroblasts has vital clinical value for the treatment of GAC.

In summary, our study showed that B7-H3 is expressed in both tumor and stromal cells of GACs. B7-H3 expressed in either tumor or stromal tissue was significantly associated with the survival and prognosis of patients. Moreover, B7-H3 expression in stromal cells was mainly detected in α-SMA-positive fibroblasts. Remarkably, GAC patients with a high stromal B7-H3 expression and a high α-SMA expression had a significantly poorer overall survival. Also, CAFs, but not NFs, highly express FAP-α and α-SMA. Furthermore, the CAF migration, invasion, and cytokine secretion abilities were significantly inhibited in the absence of B7-H3 expression. Based on our findings, we conclude that the combination of B7-H3 and α-SMA expression can be used as an indicator of a poor prognosis for GAC.

## Data Availability Statement

All datasets generated for this study are included in the article/Supplementary Material. More details are available on request from the corresponding author.

## Ethics Statement

This study was carried out in accordance with the recommendations of Clinical Research Ethics Committee of the First Affiliated Hospital of Soochow University. The protocol was approved by the Clinical Research Ethics Committee of the First Affiliated Hospital of Soochow University (approval number: 2018121). All subjects gave written informed consent in accordance with the Declaration of Helsinki.

## Author Contributions

SZ contributed to the study design, data curation, and analysis. ZL performed experiments and the writing of the original draft. MZ, TG, and QQ collected and analyzed the data. LH checked the language and format of the manuscript. LG, LC, and XZ designed the experiments and revised the manuscript. All authors approved the final version of the manuscript.

### Conflict of Interest

The authors declare that the research was conducted in the absence of any commercial or financial relationships that could be construed as a potential conflict of interest.
