# Supplementary material for: Overexpression of B7-H3 in α-SMA-Positive Fibroblasts Is Associated With Cancer Progression and Survival in Gastric Adenocarcinomas
Source: Front Oncol. 2020 Jan 10;9:1466. doi: 10.3389/fonc.2019.01466 (PMC6966326; doi:10.3389/fonc.2019.01466)
Supplement: Supplementary file 1 [file Table_1.DOCX]

Table S1 Characteristics of 268 cases of gastric adenocarcinomas

| Clinical or pathological features | Number (%) |
| --- | --- |
|  |  |
| All cases | 268 |
| Age |  |
| ﹤70 | 164 (61) |
| ≥70 | 104 (39) |
| Sex |  |
| Male | 58 (22) |
| Female | 210 (78) |
| Tumor volume (cm^3^) |  |
| ﹤5 | 186 (69) |
| ≥5 | 82 (31) |
| Tumor differentiation |  |
| Well | 6 (2) |
| Moderate | 121 (45) |
| Poor | 141 (53) |
| Tumor stage |  |
| 0 | 11 (4) |
| Ⅰ | 32 (12) |
| Ⅱ | 66 (25) |
| Ⅲ | 123 (46) |
| Ⅳ | 36 (13) |
| Tumor depth |  |
| T1 | 36 (13) |
| T2 | 34 (13) |
| T3 | 169 (63) |
| T4 | 29 (11) |
| LN involvement |  |
| N0 | 85 (32) |
| N1 | 62 (23) |
| N2 | 56 (21) |
| N3 | 65 (24) |
| Metastasis |  |
| M0 | 238 (89) |
| M1 | 30 (11) |
| Death ^a^ |  |
| No | 78 (39) |
| Yes | 120 (61) |

a: Only 198 cases of patients had information on overall survival.
