# Supplementary material for: Overexpression of B7-H3 in α-SMA-Positive Fibroblasts Is Associated With Cancer Progression and Survival in Gastric Adenocarcinomas
Source: Front Oncol. 2020 Jan 10;9:1466. doi: 10.3389/fonc.2019.01466 (PMC6966326; doi:10.3389/fonc.2019.01466)
Supplement: Supplementary file 2 [file Table_2.DOCX]

**Table S2 Primer sequences**

| primer | sequences 5'-3' |
| --- | --- |
| actin F | CATGTACGTTGCTATCCAGGC |
| actin R | CTCCTTAATGTCACGCACGAT |
| B7-H3 F | CATCACACCCCAGAGAAGCC |
| B7-H3 R | AGAGGGCCGTGCGGTTGGCA |
| VEGF F | AGGGCAGAATCATCACGAAGT |
| VEGF R | AGGGTCTCGATTGGATGGCA |
| CXCL12 F | ATTCTCAACACTCCAAACTGTGC |
| CXCL12 R | ACTTTAGCTTCGGGTCAATGC |
| IGF1 F | GCTCTTCAGTTCGTGTGTGGA |
| IGF1 R | GCCTCCTTAGATCACAGCTCC |
| PDGF F | GCAAGACCAGGACGGTCATTT |
| PDGF R | GGCACTTGACACTGCTCGT |
| FGF1 F | ACACCGACGGGCTTTTATACG |
| FGF1 R | CCCATTCTTCTTGAGGCCAAC |
| TGF-β F | GGCCAGATCCTGTCCAAGC |
| TGF-β R | GTGGGTTTCCACCATTAGCAC |
| IL-6 F | ACTCACCTCTTCAGAACGAATTG |
| IL-6 R | CCATCTTTGGAAGGTTCAGGTTG |
| TNF-α F | CCTCTCTCTAATCAGCCCTCTG |
| TNF-α R | GAGGACCTGGGAGTAGATGAG |
| IFN-γ F | TCGGTAACTGACTTGAATGTCCA |
| IFN-γ R | TCGCTTCCCTGTTTTAGCTGC |
