# Supplementary figures and images for: Overexpression of B7-H3 in α-SMA-Positive Fibroblasts Is Associated With Cancer Progression and Survival in Gastric Adenocarcinomas
Source: Front Oncol. 2020 Jan 10;9:1466. doi: 10.3389/fonc.2019.01466 (PMC6966326; doi:10.3389/fonc.2019.01466)

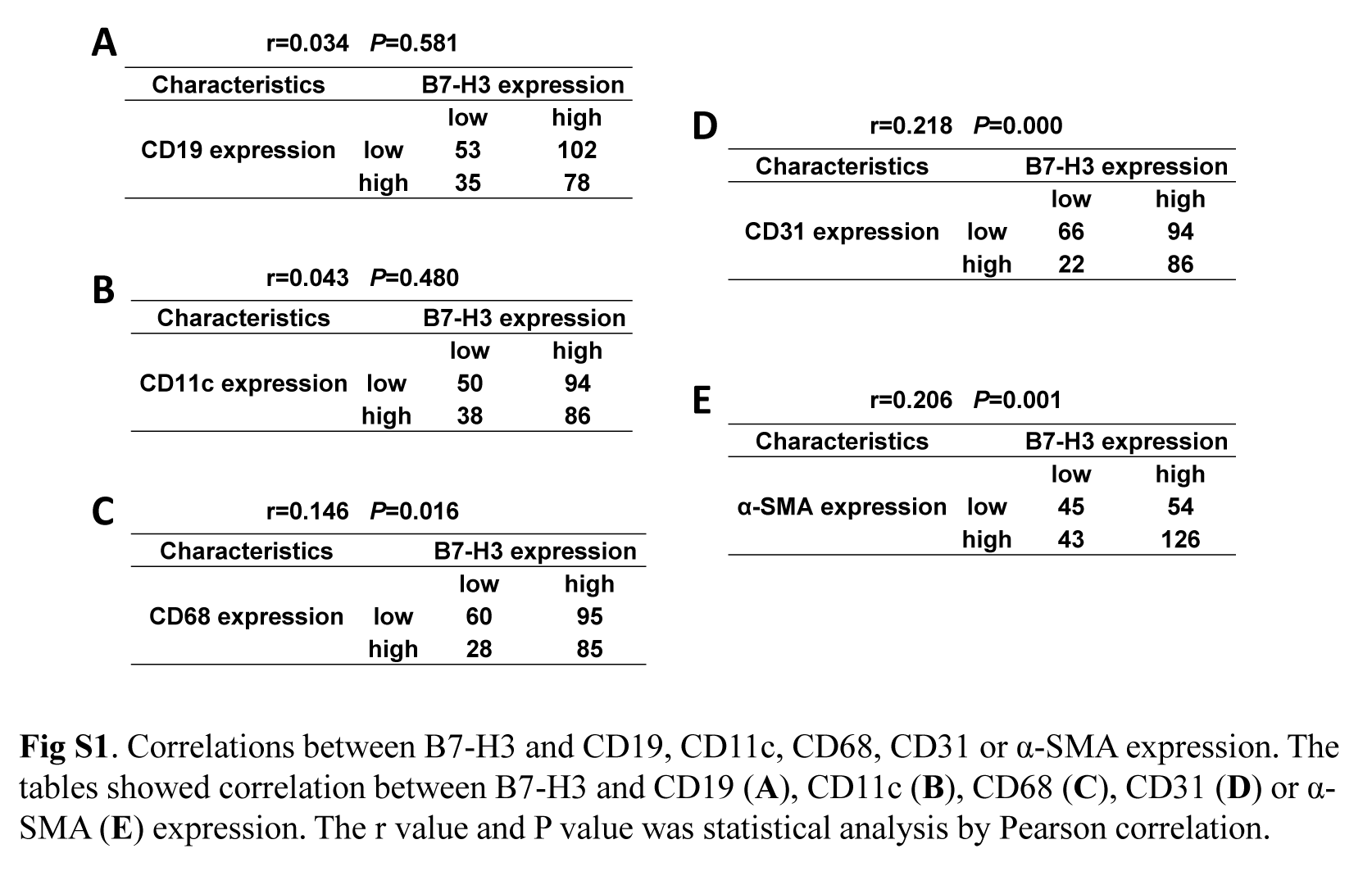

Supplement: Supplementary file 3 [file Image_1.TIF]
